# Dietary long-chain omega 3 fatty acids modify sphingolipid metabolism to facilitate airway hyperreactivity

**DOI:** 10.1038/s41598-022-21083-w

**Published:** 2022-11-17

**Authors:** Andrea Heras, Rika Gomi, Madeline Young, Chuchun L. Chang, Emily Wasserman, Anurag Sharma, Wenzhu Wu, Jinghua Gu, Uthra Balaji, Rachel White, Perdita Permaul, Ibrahim Janahi, Tilla S. Worgall, Stefan Worgall

**Affiliations:** 1 Department of Pediatrics, Weill Cornell Medicine, 413 East 69th Street, Room 1200, New York, NY 10021 USA; 2 Institute of Human Nutrition/Department of Pediatrics, Columbia University Medical Center, New York, NY USA; 3 Weill Cornell Medicine, Drukier Institute for Children’s Health, New York, USA; 4 Department of Pediatrics, Sidra Hospital, Doha, Qatar; 5 Department of Pathology and Cell Biology, Columbia University Medical Center, New York, USA; 6 Department of Genetic Medicine, Weill Cornell Medicine, New York, USA

**Keywords:** Sphingolipids, Asthma

## Abstract

Omega-3 polyunsaturated fatty acids (n-3 PUFAs) are essential nutrients that can affect inflammatory responses. While n-3 PUFAs are generally considered beneficial for cardiovascular disease and obesity, the effects on asthma, the most common inflammatory lung disease are unclear. While prenatal dietary n-3 PUFAs decrease the risk for childhood wheezing, postnatal dietary n-3 PUFAs can worsen allergic airway inflammation. Sphingolipid metabolism is also affected by dietary n-3 PUFAs. Decreased sphingolipid synthesis leads to airway hyperreactivity, besides inflammation, a cardinal feature of asthma, and common genetic asthma risk alleles lead to lower sphingolipid synthesis. We investigated the effect of dietary n-3 PUFAs on sphingolipid metabolism and airway reactivity. Comparing a fish-oil diet with a high n-3 PUFA content (FO) to an isocaloric coconut oil-enriched diet (CO), we found an n-3 PUFA-dependent effect on increased airway reactivity, that was not accompanied by inflammation. Lung and whole blood content of dihydroceramides, ceramides, sphingomyelins, and glucosylceramides were lower in mice fed the n-3 PUFA enriched diet consistent with lower sphingolipid synthesis. In contrast, phosphorylated long chain bases such as sphingosine 1-phosphate were increased. These findings suggest that dietary n-3 PUFAs affect pulmonary sphingolipid composition to favor innate airway hyperreactivity, independent of inflammation, and point to an important role of n-3 PUFAs in sphingolipid metabolism.

## Introduction

Polyunsaturated fatty acids (PUFAs), essential nutrients with a multitude of biological effects mainly related to growth and metabolism, are actively incorporated as acyl chains into cell membrane lipids, including sphingolipids, and can affect membrane scaffold formation, energy storage and signal transduction by lipid mediators^1^. N-3 PUFAs have anti-inflammatory effects^2^ which attenuate systemic inflammation associated with obesity and cardiovascular disease^3,4^. N-3 PUFAs may also be beneficial in asthma as: (1) Exhaled breath condensates of asthmatic individuals contained lower levels of a n-3 PUFA docosahexaenoic acid derivative^5^; and decreased airway reactivity inflammation with allergic sensitization can be achieved (2) by oral or aerosolized administration n-3 PUFAs or derivatives^5–10^; and (3) by endogenously increasing n-3 PUFAs in transgenic mice expressing a n-3 fatty acid desaturase^11^. In contrast, exacerbation of inflammation by n-3 PUFAs has been seen with allergic airway and intestinal inflammation^12–14^ and infection^15–17^ models.

Polymorphisms within the 17q21 chromosomal region that increase expression of the sphingolipid synthesis inhibitor ORMDL3 are linked to childhood asthma^18–20^ and obesity^21^. ORMDL3 inhibits serine palmitoyl transferase (SPT), the rate-limiting enzyme in de novo sphingolipid synthesis^22,23^. ORMDL3-overexpressing mice as well as knockdown or pharmacological inhibition of SPT lead to decreased lung sphingolipid levels and innate airway hyperreactivity^24,25^. We investigated the effects of n-3 PUFAs on sphingolipid metabolism and airway reactivity, a key characteristic of all asthma types, by comparing mice that were fed for more than two months with either the n-3 PUFA-enriched FO diet or the coconut-oil enriched CO diet.

## Results and discussion

**Figure 1:**
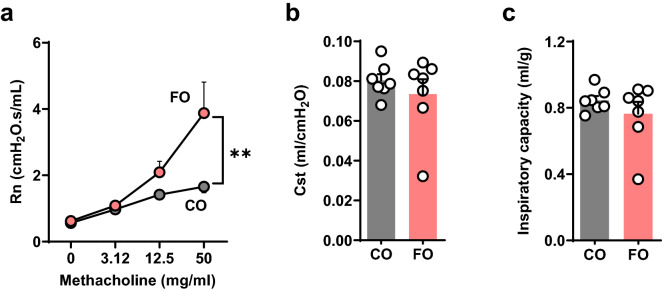
N-3 PUFA enriched diet induces innate airway hyperreactivity. BALB/c mice were fed a fish oil diet (FO) or an isocaloric control diet (CO) for 8 weeks. Pulmonary function testing was performed on anaesthetized and tracheotomized mice using a mouse pulmonary function system (Scireq). (**a**) Airway resistance (Rn) with increasing doses of methacholine. (**b**) Static compliance (Cst). (**c**) Inspiratory capacity (IC). Data are means ± SEM of 7 animals per group. **p < 0.001. Shown are results of ANOVA (Rn) or unpaired T-test (Cst, IC).

Both FO and CO fed mice showed similar weight gain (Supplemental Fig. 1a) and similar food consumption (Supplemental Fig. 1b). We next evaluated the effects of the two high fat diets on plasma sphingolipid levels. FO resulted in higher plasma levels of all sphingoid long chain bases (Supplemental Fig. 2a), dihydroceramides (Supplemental Fig. 2b) and sphingomyelins (Supplemental Fig. 2c). Plasma ceramide levels were lower with FO compared to CO (Supplemental Fig. 2d), mostly due to ceramide C24:1 that itself was not significantly different between the two groups (p < 0.087). As decreased levels of sphingolipids that are generated mainly via de novo synthesis via SPT such as sphingosine and dihydroceramides are associated with increased airway reactivity^25–27^, we hypothesized that the FO diet would lead to lower airway reactivity compared to CO. Surprisingly, airway reactivity (Rn) in response to methacholine was increased in mice fed FO (Fig. 1a). Lung compliance (Fig. 1b) and inspiratory lung capacity (Fig. 1c) were similar in both groups.

A similar pattern was seen in C57BL/6 mice (Supplemental Fig. 3a–c). This suggests that the PUFA-enriched diet induced a lung phenotype of innateairway hyperreactivity without other functional impairment typically associated with chronic inflammation or fibrosis. BALB/C and C57BL/6 were selected to assess if the innate airway hyperreactivity is independent of the genetic inflammatory haplotype^28^ with Th1- and M1-dominant responses in C57BL/6, and Th2- and M2-dominant responses in BALB/c mice, respectively^29^. The effect was only evaluated in female mice. Increased innate airway reactivity may likely be also seen with male mice, as sphingolipid-associated innate airway hyperreactivity we independent of sex and also equally seen in both strains^30^.

Asthmatic airway hyperreactivity is usually associated with airway inflammation. N-3 PUFAs in dietary fish oil supplements are known to effect airway inflammation in asthma, but the results have not been consistent^6–8,11–13,31–33^.

**Figure 2:**
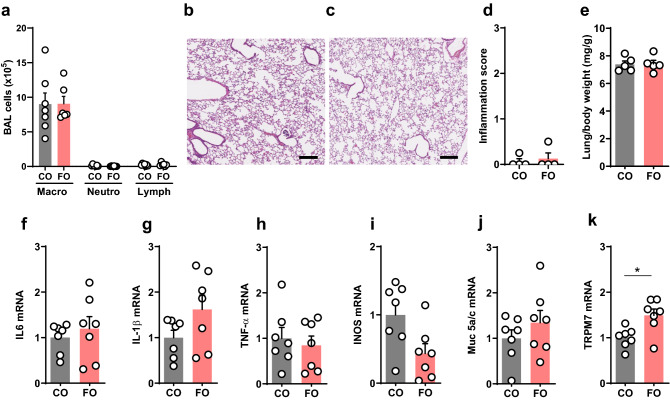
N-3 PUFA-enriched diet does not induce pulmonary inflammation. Analyses of lung and broncho-alveolar lavage fluid (BAL) from BALB/c mice that were fed a fish oil diet (FO) or an isocaloric control diet (CO) for 8 weeks. (**a**) Cytospin analysis of BAL cells. (**b**, **c**) Lung histology of CO (**b**) and FO (**c**) mice. Shown are representative lung sections stained with H&E, bar equals 250 μm. (**d**) Inflammatory score of histological sections. (**e**) Lung wet weights relative to body weight. (**f**–**k**) Expression of inflammatory markers in lung analyzed by Real-Time PCR: (**f**) IL-6. (**g**) IL-1β. (**h**) TNF-α (**i**) INOS. (**j**) Muc5AC; and (**k**) TRPM7. Data are means ± SEM of 5 animals per group. *p < 0.05. **p < 0.001, ***p < 0.0001. Shown are results of unpaired T-tests.

The cellular composition of the BAL, a solid parameter to assess airway inflammation relevant to asthma, showed a normal macrophage predominance in both groups (Fig. 2a). Likewise, no lung or airway inflammation was visible histologically (Fig. 2b–d), and wet lung weights were similar in both groups (Fig. 2e). Expression of the inflammatory factors IL-6 (Fig. 2f), IL-1b (Fig. 2g), TNF-a (Fig. 2h), inducible nitric oxide synthase (Fig. 2i), and the mucus gene Muc5a/c (Fig. 2j) were similar in both groups. Interestingly, expression of the calcium and magnesium transporter TRPM7, which is increased in SPT-deficient mice with increased airway reactivity^25^, was increased in the FO group (Fig. 2k). This suggests that the higher airway reactivity in the FO-fed mice was not induced by lung inflammation. It further signifies that allergic sensitization, which was used in all prior studies assessing the effects of n-3 PUFAs on airway inflammation and reactivity^11–13^, was not required for the innate airway hypereactivity phenotype.

**Figure 3:**
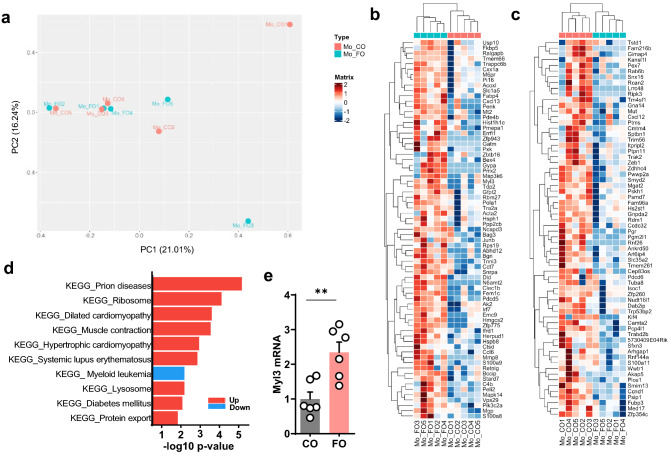
Lung transcriptomes from mice fed with n-3-PUFA diet. Clustering of lung transcriptomes and differential gene expression from FO or CO fed mice. RNA-sequencing was performed on RNA isolated from lung tissue after 8 weeks. (**a**) Principal component analysis (PCA) from whole lung. Differential gene expression analysis, comparison is of FO and CO groups. (**b**) Genes relatively upregulated in the FO group. (**c**) Genes relatively downregulated in the FO group. (**d**) Summary of KEGG pathways associated with differentially expressed genes (p < 0.05), comparison is of FO vs CO groups with upregulated in red and downregulated pathways in blue. (**e**) Expression of Myl3 in lungs from mice from independent experiments by Real-Time PCR. **p < 0.001 (unpaired T-test).

To further assess the effects of the n-3 PUFAs on pulmonary gene expression, lung transcriptomes were analyzed by RNA-seq. Global gene expression was similar between both groups (Fig. 3a). Differential gene expression (DEG) analysis revealed only 137 DEG genes when comparing FO and CO groups (Fig. 3b,c). Among those, numerous genes arerelated to smooth muscle cell contractility and pro- and anti-inflammatory effects relevant to asthma (Supplemental Table 2). The majority of genes related to smooth muscle cell contraction or growth were higher expressed in the FO group, whereas there was an even pro- and anti-inflammatory gene expression effect of FO. To put the overall gene expression analysis in biological context, gene set enrichment analysis was performed for KEGG (Kyoto Encyclopedia of Genes and Genomes) pathways. KEGG pathway analysis showed upregulation of two cardiac muscle pathways and one contraction pathway (p < 0.05) in the FO group (Fig. 3d). These three pathways consist of overlapping genes, e.g. 31 out of 34 for dilated and hypertrophic cardiomyopathy, 17 out of 30 for muscle contraction and either dilated or hypertrophic cardiomyopathy. All three pathways contain numerous genes that also play a role in smooth muscle cell contraction (dilated cardiomyopathy: 22 out of 34 genes; cardiac muscle contraction: 15 out 30 genes; hypertrophic cardiomyopathy: 21 out of 34 genes), suggesting a relevance for airway smooth muscle cell contraction. All three also included increased expression of myosin light chain 3 (Myl3; Fig. 3b). Realtime PCR confirmed increased lung expression of Myl3 in the FO group (Fig. 3e).

Expression of Myl3 is high in cardiac muscle^34^, it is also expressed in human lung smooth muscle and fibroblasts^35^ and in mouse lung^36^. Myl3 encodes a myosin light chain and in airway smooth muscle, signaling through myosin light chain kinase is critical for contractile function^37^, It is unknown what functional role Myl3 has in airway smooth muscle cells. Interestingly and relevant to our manuscript, a large European cross-trait genome-wide association study to identify shared genetic components between obesity-related traits and specific asthma subtypes identified 34 shared loci^38^. Among those were acyl-coenzyme A oxidase-like (ACOXL) and myosin light chain 6 (Myl6). Those two genes were then also confirmed by RNA sequencing from lungs of diet -induced-obese versus control mice^38^, an established model for obesity-induced innate hyperresponsiveness^39^. While Myl6 was not identified in our screen, it is similar to Myl3, and ACOXL is also one of the genes upregulated in the fish oil group (Fig. 3c), suggesting similarities between asthma models of innate airway hyperreactivity and the dietary effects of n-3 PUFAs. Gene ontology (GO) pathway analysis was also performed and showed upregulation of some inflammatory pathways in the FO group, though with a p-value < 0.1 (Supplemental Fig. 4). Increased oxidative stress pathways were only identified under less stringent statistical criteria and showed some upregulation of the GO pathways positive regulation of response to oxidative stress (adjusted p-value = 0.6), negative regulation of oxidative stress-induced neuron intrinsic apoptotic pathway (adjusted p-value = 0.61), oxidative demethylation (adjusted p-value = 0.63), and positive regulation of oxidative stress-induced intrinsic apoptotic signaling pathway (adjusted p-value = 0.65), suggesting no clear positive and or negative effect on oxidative stress pathways with n-3 PUFAs as a potential cause for increased airway reactivity. Genes within the sphingolipid biosynthesis and acute inflammatory response pathways were similarly expressed in the lungs from both groups (Supplemental Fig. 5). Overall, while the RNA seq data showed some lung gene expression differences between the CO and FO, the overall number of differentially expressed genes was small, but did include genes to muscle contraction, such as myl3. Interestingly, expression of the magnesium transporter TRPM7 that has been associated with increased airway contractility in SPT-deficient mice^25^, was higher with the FO diet, suggesting decreased sphingolipid synthesis without changes in transcription genes for enzymes within this pathway. This data reiterates that pulmonary inflammation is not the major factor for the increased airway reactivity induced by the FO diet. As decreased pulmonary and blood cell sphingolipid synthesis is associated with increased airway reactivity in the absence of inflammation^25,40^, we assessed lung and blood cell sphingolipid levels at the time of the pulmonary function testing.

**Figure 4:**
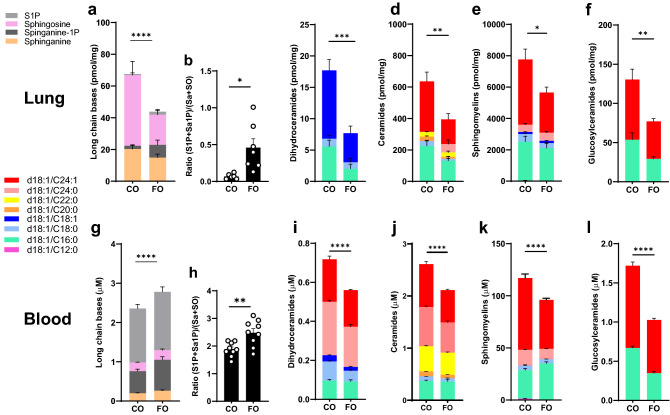
N-3 PUFA enriched diet decreases ceramides and sphingomyelins in lungs and blood cells. Sphingolipids in lung (**a**–**f**) and whole blood (**g**–**l**) from BALB/c mice that were fed either FO or CO diet for 8 weeks. (**a**, **g**) Long chain bases. (**b**, **h**) Ratios of the two phosphorylated long chain bases sphingosine-1P (S1P) and sphinganine-1P (Sa1P) to sphinganine and sphingosine. (**c**, **i**) Dihydroceramides. (**d**, **j**) Ceramides. (**e**, **k**) Sphingomyelins. (**f**, **l**) Glucosylceramides. Data are representative of 3 independent experiments with 5–6 mice per group. *p < 0.05. **p < 0.001, ***p < 0.0001, ****p < 0.00001 (two-way ANOVA with Tukey’s multiple comparisons test).

In contrast to the plasma levels, lung long chain bases (Fig. 4a), dihydroceramides (Fig. 4c), ceramides (Fig. 4d), sphingomyelins (Fig. 4e), and glucosylceramides (Fig. 4f) were decreased in the FO compared to the CO group. Sphingolipid composition in blood cells, a parameter of cellular sphingolipid composition relevant to asthma^30^, also showed lower levels of dihydroceramides (Fig. 4i), ceramides (Fig. 4j), sphingomyelins (Fig. 4k), and glucosylceramides (Fig. 4l) with the FO diet. The sum of long chain bases in the blood was overall increased in the FO mice compared to CO (Fig. 4g), mainly due to sphinganine-1P (p = 0.0054). Plasma sphingolipid levels, with the exception of ceramides, were higher with FO diet compared to CO diet, in contrast to lung and whole blood where most sphingolipids were lower. Whole blood sphingolipids seem to be better reflect tissue sphingolipid levels compared serum or plasma levels. Genetically decreased sphingolipid synthesis in children with asthma was detectable in whole blood and PBMCs, and reflected levels in airway epithelial cells^30^. There is limited information on the effects of n-3 PUFAs on blood sphingolipidomes in humans. Targeted lipidomics for glycerophospholipids and sphingolipids of healthy individuals in plasma following dietary supplementation with n-3 PUFAs for three weeks showed a trend for increased ceramides and dihydrocermides^41^. Dietary supplementation of healthy subjects with EPA and DHA for three months reduced ceramides in VLDL and increased sphingomyelin in LDL^42^. Our data suggests that n-3 PUFA-enriched diets can lower tissue and cell sphingolipids. However, ratios of the phosphorylated long chain bases S1P and Sa-1P to sphingosine and sphinganine were higher with FO in blood (Fig. 4b) and lung (Fig. 4h). This finding is in contrast to SPT-deficient mice^25^ and suggests an effect of the n-3 PUFAs on increasing sphingosine kinase activity or decreasing degradation by sphingosine phosphate lyase. Increased S1P is associated with increased airway reactivity and asthma, as S1P can directly contract airway smooth muscle through signaling via S1P receptors^43^ and can also lead to airway remodeling^44,45^. There were no signs for airway remodeling histologically or functionally with normal compliance and absence of fixed obstruction in the lung function studies. While the lung and blood sphingolipid profiles seen with FO do not suggest a primary effect on sphingolipid de novo synthesis, both increased S1P and decreased dihydroceramides could have been instrumental for the hyperreactivity. Effects on membrane lipid rafts could be potential mechanisms for the n-3 PUFA induced changes of cellular sphingolipids on airway reactivity^46^. Rafts, usually enriched with ceramides, sphingomyelins and glucosphingolipids^47^, all lower in blood cells and lung with FO, are involved in the clustering of membrane signaling proteins to facilitate cell signaling, a process that is perturbed by n-3 PUFAs^48–50^.

Overall, the study shows a strong effect of n-3 PUFAs in a hypercaloric diet on blood and tissue sphingolipids. The combination of decreased sphingolipids and increased circulating S1P may provide the functional link for enhanced airway reactivity in the absence of inflammation. Further studies need to assess n-3 PUFA-induced disturbances of lipid rafts and effects on signaling, especially of pathways critical for smooth muscle cell contraction.

## Materials and methods

All methods were carried out in accordance with relevant guidelines and regulations, and are reported in accordance with ARRIVE guidelines.

### Mice and diets

All animal studies were conducted under protocols approved by the Institutional Animal Care and Use Committee of Weill Cornell Medicine. Female BALB/c and C57Bl/6 mice were purchased at 4–6 weeks of age from Jackson Laboratory and housed under specific pathogen-free conditions. Following 1 week acclimatization, mice were fed a high-fat, semipurified diet (total 19% fat, 0.2% cholesterol, w/w) enriched in either n-3 PUFAs (91% menhaden fish oil and 9% corn oil; Harlan Teklad; TD. 07500; FO) or saturated fat (75% saturated fat from coconut oil, 17% monounsaturated fat from olive oil, and 8% polyunsaturated fat from corn oil; Harlan Teklad; TD. 08081; CO) for 8–11 weeks. Both diets have been used to assess the attenuating effects of PUFAs on atherosclerosis and inflammation^51^. Detailed composition of the diets is provided in Supplemental Table 1. Weight and food intake were checked weekly. After 8–10 weeks the animals underwent pulmonary function testing and were sacrificed.

### Blood and lung sphingolipid analyses

Sphingolipids were quantified in plasma, dried blood spots, and homogenized lung by high pressure liquid chromatography electrospray ionization tandem mass spectrometry (HPLC–MS/MS) using minor modification of a described method^52^. The method is validated for five dihydroceramides: (d18:0/16:0 d18:0/18:0, d18:0/18:1, d18:0/24:0, d18:0/24:1), six ceramides (d18:1/C16:0, d18:1/C18:0, d18:1/C20:0, d18:1/C22:0, d18:1/C24:0, d18:1/C24:1), two glucosylceramides (GlcCer d18:1/C18:0, GlcCer d18:1/C24:1), five sphingomyelins (SM d18:1/C12:0, SM d18:1/C16:0, SM d18:1/C18:0, SM d18:1/C18:1, SM d18:1/C24:1), and the four long-chain bases sphinganine (SA d18:0), sphingosine (SO d18:1), sphinganine-1-phosphate (Sa-1-P d18:0), sphingosine-1-phosphate (S1P d18:1). 25 ul plasma, whole blood and lung homogenate were extracted by vortexing overnight in 900 ul dichloromethane/methanol (1:1) with addition of internal standard (*N*-lauroyl-d-erythro-sphingosylphosphorylcholine). After centrifugation to precipitate cell debris, an aliquot was transferred into an Agilent 1200 HPLC (Agilent Poroshell 120 column) linked to an Agilent 6430 triple quadrupole mass spectrometer. Mobile phase A consisted of methanol/water/chloroform/formic acid (55:40:5:0.4 v/v); Mobile phase B consisted of methanol/acetonitrile/chloroform/formic acid (48:48:4:0.4 v/v). After pre-equilibration for 6 s, the gradient was increased gradually to 60% mobile phase B and 100% mobile phase B was held for 1.9 min. With a flow rate is 0.6 mL/min, the duration of the entire run was 9.65 min. We used the Mass Hunter optimizer and pure synthetic standards (Avanti Polar Lipids) to determine optimum fragmentation voltage, precursor/product ions and m/z values. Peak calls and abundance calculations were obtained with MassHunter Workstation Software Version B.06.00 SP01/Build 6.0.388.1 (Agilent). Final concentrations were calculated from a standard curve for each sphingolipid run in parallel.

### Lung mechanics and airway reactivity

Mice were anaesthetized with pentobarbital (100 mg/kg; American Pharmaceutical Partners), tracheostomized and mechanically ventilated using a computer-controlled animal ventilator (FlexiVent, SCIREQ). Respiratory mechanics were analyzed using the FlexiVent software as previously described^25,53^. Static compliance was determined using the Salazar-Knowles equation to the plateau pressure measurements obtained between total lung capacity and functional residual capacity. Broadband forced oscillations were applied to determine Newtonian (airway) resistance (Rn) using a constant phase model. Rn was also assessed following increasing doses of methacholine (3.125, 12.5 and 50 mg/ml) to quantify airway reactivity.

### Lung inflammation

Bronchoalveolar lavage (BAL) fluid was collected by three intratracheal instillations of PBS, 0.5 mM EDTA (total 3.5 ml), centrifuged at 450 G for 7 min at 4 °C, and cells were resuspended in PBS. Cell differentials were determined by Giemsa stain on cytospin preparations. Cell viability was determined by trypan blue exclusion. RNA was extracted from homogenized lung tissue using TRIzol (Invitrogen). Following reverse transcription, TaqMan Gene expression assays were performed using probes for IL6 (Mm00445197_m1), IL1-β (Mm00445197_m1), TNF-α (Mm00445197_m1), iNOS (Mm01208059_m1), MUC5ac (Mm01276718_m1), TRPM7 (Mm00457998_m1), S100a9 (Mm00656925_m1), Myl3 (Mm00491655_m1); all from ThermoFisher Scientific). The mRNA levels were quantified using the ∆∆Ct method and normalized to expression of eukaryotic 18S rRNA endogenous control (4352930E, Applied Biosystems). For lung histology, lungs were inflated with 4% paraformaldehyde at 25 cm H_2_O for 16–24 h and 5 μm paraffin sections were stained with H&E. Histological scoring for inflammation was performed based on cellular infiltration in the alveolar parenchyma away from major vessels, the perivenular regions, and the bronchoarterial regions using a 0–3 severity score. A score of 0 indicates no inflammatory cells, a score of 1 represents occasional cuffing by inflammatory cells, a score of 2 indicates a thin layer (1–5 cells thick) of inflammatory cells, and a score of 3 indicates a thick layer (more than 5 cells thick) of inflammatory cells. Combined grading was based on the most severely inflamed section on each slide.

### Lung RNA sequencing

RNA was extracted using Promega Maxwell 16 MDx instrument, (Maxwell 16 LEV simplyRNA Tissue Kit). Specimens were prepared for RNA sequencing using TruSeq RNA Library Preparation Kit v2 or riboZero as previously described^54^. RNA integrity was verified using the Agilent Bioanalyzer 2100 (Agilent Technologies). cDNA was synthesized from total RNA using Superscript III (Invitrogen). Sequencing was then performed on GAII, HiSeq 2000, or HiSeq 2500, as single end reads^55,56^. All reads were independently aligned with STAR_2.4.0f1^57^ for sequence alignment against the mm10 murine genome. SAMTOOLS v0.1.19 was used for sorting and indexing reads^58^. Gene counts from htseq-count^59^ and DESeq2 bioconductor package^60^ were used to identify differentially expressed genes (DEGs) setting a threshold of nominal p value less than 0.05. For biological context, GeneSet Enrichment Analysis (GSEA) was performed for KEGG pathways^61–63^ and Gene Ontology biological processes (GO BP) obtained from molecular signature database using msigdbr package^64^. Over-representation analysis was performed using the fgsea package. Pheatmap and ggplot2 packages were used to generate visualization plots^65^. For biological context, GeneSet Enrichment Analysis (GSEA) was performed for KEGG pathways and Gene Ontology biological processes (GO BP) obtained from molecular signature database using msigdbr package^64^.

### Statistics

The results are presented as mean ± SEM. We first used the Shapiro–Wilk normality test for all data to confirm that our data were normally distributed. Comparisons between two groups were conducted by unpaired t-test. Comparisons of the pulmonary function tests were conducted by one-way ANOVA with Dunnett post-hoc test or one-way ANOVA with Tukey’s comparisons test. P values for two-way ANOVA were adjusted to account for multiple comparisons. For all tests, differences were considered significant when p < 0.05 and four significance levels are indicated as follows: *p < 0.05, **p < 0.01, ***p < 0.001, ****p < 0.0001. Graph-Pad Prism™ vs. 8.2 was used for all statistical analyses.

## Supplementary Information

Supplementary Information.

## Data Availability

The datasets generated and/or analysed during the current study are available in the Gene Expression Omnibus (GEO) repository (https://www.ncbi.nlm.nih.gov/geo/query/acc.cgi?acc=GSE203418).
