# Supplementary material for: Dietary long-chain omega 3 fatty acids modify sphingolipid metabolism to facilitate airway hyperreactivity
Source: Sci Rep. 2022 Nov 17;12:19735. doi: 10.1038/s41598-022-21083-w (PMC9672127; doi:10.1038/s41598-022-21083-w)
Supplement: Supplementary file 1 — Supplementary Information. [file 41598_2022_21083_MOESM1_ESM.docx]

**Supplementary Information for:**

**Dietary long-chain omega 3 fatty acids modify sphingolipid metabolism to facilitate airway hyperreactivity**

Andrea Heras, Rika Gomi, Madeline Young, Chuchun L. Chang, Emily Wasserman, Anurag Sharma, Rohan Bareja, Jinghua Gu, Uthra Balaji, Rachel White, Ibrahim Janahi, Tilla S. Worgall, Stefan Worgall

**This file includes:**

Supplemental Figures S1 to S5

Supplemental Table

**Supplemental Figures**

**
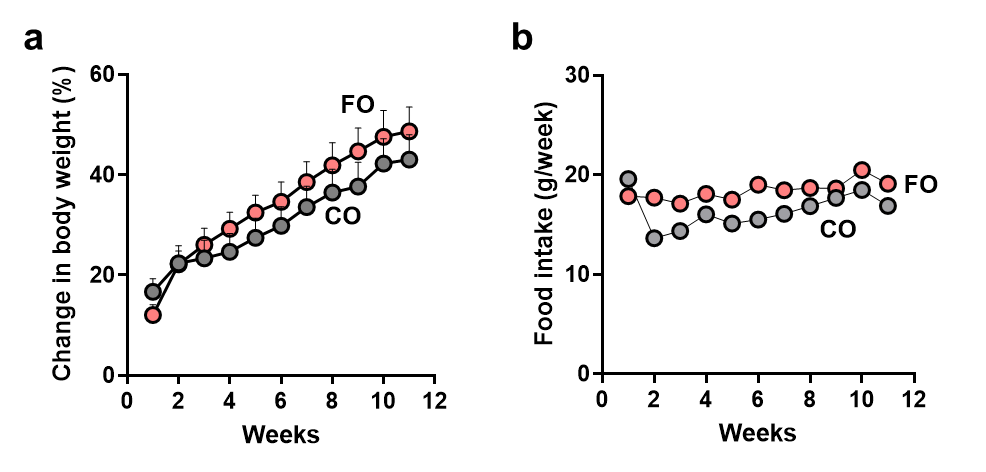
**

**Supplemental Fig. 1.** N-3 PUFA- and CO enriched high fat diets increase body weight.

BALB/c mice were fed a fish oil diet (FO), an isocaloric high fat coconut oil diet (CO) for 12 wk. Body weight and food consumption were monitored weekly. **a,** Body weight. **b,** Food consumption. Data are means +/- SEM of 5-7 animals per group. *p<0.05. ** p<0.001. Shown are results of ANOVA with Tukey’s multiple comparisons test.

**
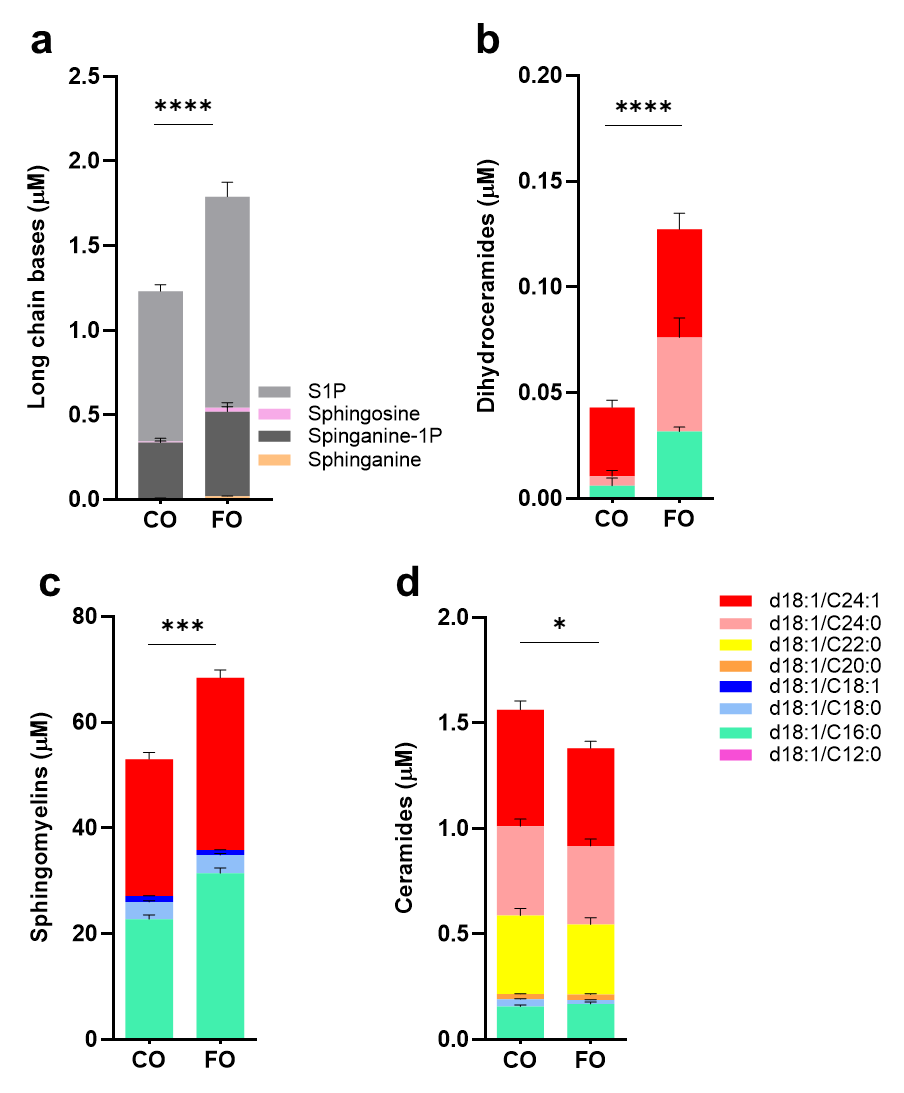
**

**Supplemental Fig. 2.** N-3 PUFA-enriched diet increases plasma sphingolipids. BALB/c mice were fed a fish oil diet (FO) or an isocaloric control diet (CO) for 8 wk. Plasma sphingolipid levels were analyzed by HPLC-MS/MS. **a,** Long-chain bases. **b,** Dihydroceramides. **c,** Ceramides. **d,** Sphingomyelins. Data are representative of 3 independent experiments with 5-7 mice per group. Data are means +/- SEM. *p<0.05. *** p<0.0001, **** p<0.00001 (two-way ANOVA with Tukey’s multiple comparisons test).

**
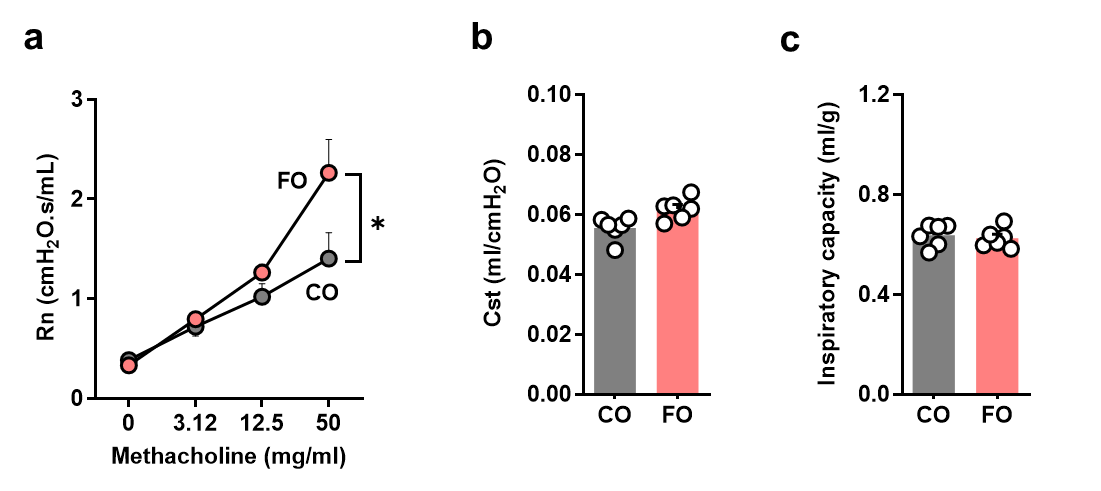
**

**Supplemental Fig. 3.** N-3 PUFA enriched diet leads to innate airway hyperreactivity in C57BL/6 mice C57BL/6 mice were fed a fish oil diet (FO) or an isocaloric control diet (CO) for 8 wk. Pulmonary function testing was performed on anaesthetized and tracheotomized mice using mouse pulmonary function system (Scireq). **a,** Airway resistance (Rn) with increasing doses of methacholine. **b,** Static compliance (Cst). **c,** Inspiratory capacity (IC). Data are means +/- SEM of 6 animals per group. ** p<0.001. Shown are results of ANOVA (Rn) or unpaired T-test (Cst, IC).


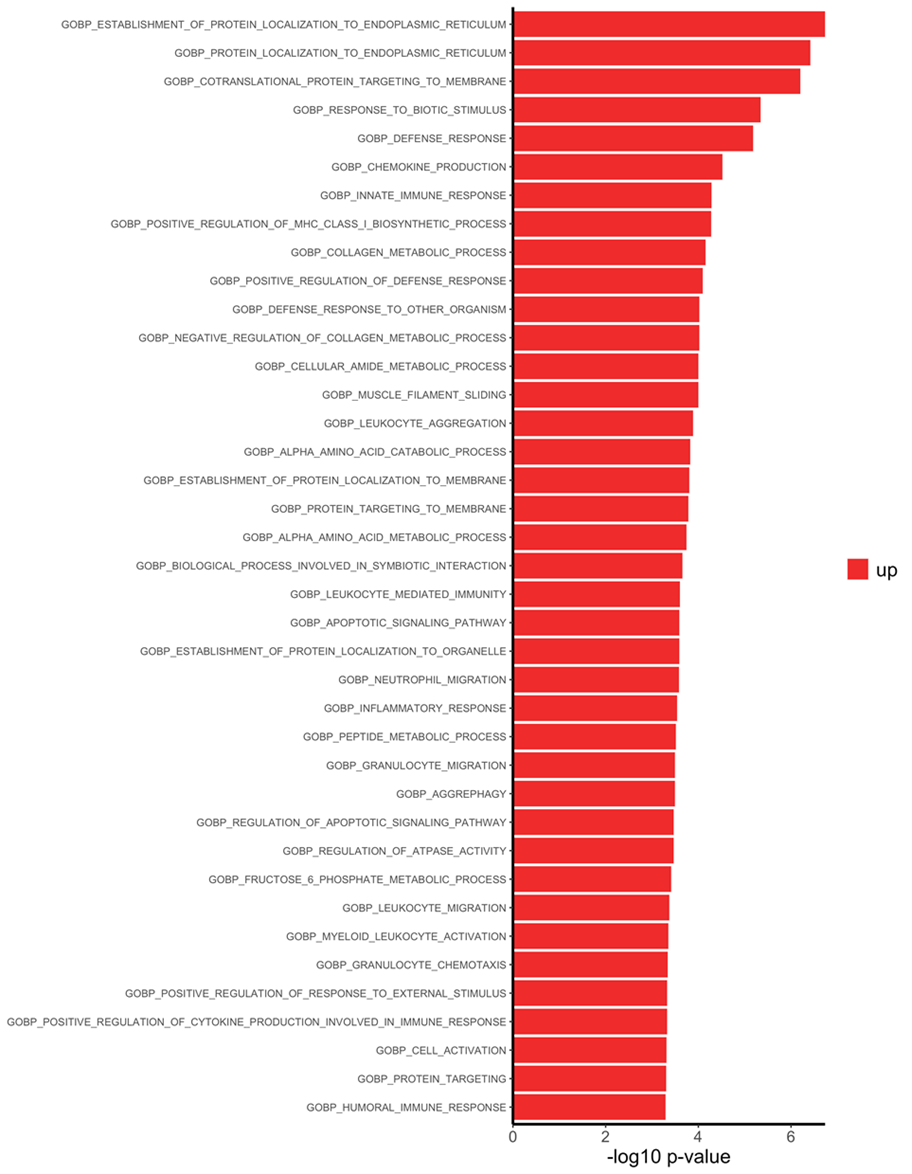


**Supplemental Fig. 4.** Summary of Gene Ontology Pathway analysis. Comparison is of FO vs CO groups, adjusted p-value < 0.1 for differentially expressed genes. Upregulated pathways in red.

**Supplemental Fig. 5.** Gene ontology enrichment analysis of differentially expressed inflammatory and sphingolipid synthesis genes. **a,** Acute inflammatory response genes (GO:0002526). **b,** Genes within the sphingolipid synthesis pathway (GO: 0030148). Shown are results of hierarchical clustering of differentially expressed genes of FO or CO fed mice. Data represents 5 mice per group.

**Supplemental Table**

| *Continued*  **Vitamins (g/kg)** | **TD08081**  **(CO)** | **TD07500**  **(FO)** |
| --- | --- | --- |
| Calcium pantothenate | 0.02 | 0.02 |
| Pyridoxine HCl | 0.008 | 0.008 |
| Thiamin (81%) | 0.007 | 0.007 |
| Riboflavin | 0.007 | 0.007 |
| Folic acid | 0.002 | 0.002 |
| Biotin | 0.0002 | 0.0002 |
| Vitamin B12 (0.1% in mannitol) | 0.03 | 0.03 |
| Vitamin E, DL-alpha tocopherol acetate (500 IU/g) | 0.18 | 0.18 |
| Vitamin A palmitate (5x10^5^ IU/g) | 0.01 | 0.01 |
| Vitamin D3, cholecalciferol (5x10^5^ IU/g) | 0.002 | 0.002 |
| Vitamin K1, phylloquinone | 0.0009 | 0.0009 |
| Sucrose, fine ground | 11.7 | 11.7 |
|  |  |  |
| **Sphingolipids** (pmol/mg)**:** |  |  |
| ***Dihydroceramides, all:*** | 0.07 | 0.20 |
| d18:0/16:0 | 0.05 | 0.07 |
| d18:0/18:0 | 0.01 | 0.04 |
| d18:0/18:1 | 0.01 | 0.07 |
| d18:0/24:0 | 0.00 | 0.01 |
| d18:0/24:1 | 0.00 | 0.01 |
| ***Ceramides, all:*** | 0.39 | 1.24 |
| d18:1/C16:0 | 0.19 | 0.39 |
| d18:1/C18:0 | 0.18 | 0.63 |
| d18:1/C20:0 | 0.00 | 0.01 |
| d18:1/C22:0 | 0.01 | 0.08 |
| d18:1/C24:0 | 0.01 | 0.08 |
| d18:1/C24:1 | 0.00 | 0.05 |
| ***Sphingomyelins, all:*** | 12.08 | 14.05 |
| SM d18:1/C12:0 | 0.04 | 0.08 |
| SM d18:1/C16:0 | 0.24 | 0.28 |
| SM d18:1/C18:0 | 9.7 | 10.77 |
| SM d18:1/C18:1 | 0.64 | 0.50 |
| SM d18:1/C24:0 | 0.29 | 0.25 |
| SM d18:1/C24:1 | 1.17 | 2.16 |
| LysoSM d18:1 | 0.00 | 0.01 |
| ***Glucosylceramides, all:*** | 1.61 | 2.11 |
| GlucCer(d18:2/16:0) | 0.94 | 1.47 |
| GlucCer(d18:2/24:1) | 0.62 | 0.58 |
| GlucSO d18:1 | 0.05 | 0.06 |
| ***Long-chain bases, all:*** | 0.06 | 0.22 |
| SA d18:0 | 0.01 | 0.06 |
| SO d18:1 | 0.04 | 0.14 |
| PhytoSO d18:0 | 0.01 | 0.02 |

|  | **TD08081**  **(CO)** | **TD07500**  **(FO)** |
| --- | --- | --- |
| Protein (%) | 17.7 | 17.7 |
| Carbohydrates (%) | 39.3 | 39.3 |
| Fat (%) | 18.3 | 18.7 |
| Cholesterol (%) | 0.20 | 0.12 |
| Kcal/g | 4 | 4 |
| Ratio of omega 6 to omega 3 PUFAs | 46 | 0.3 |
| Saturated fatty acids (%) | 75 | 29 |
| Monounsaturated fatty acids (%) | 17 | 25 |
| Polyunsaturated fatty acids (%) | 8 | 46 |
| DHA (% of total fatty acids) | 0 | 8-10 |
| EPA (% of total fatty acids) | 0 | 12-15 |
|  |  |  |
| **Calories provided by:** |  |  |
| Protein, % | 17.9 | 17.9 |
| Fat (ether extract), % | 42.4 | 39.7 |
| Carbohydrates, % | 39.7 | 42.4 |
|  |  |  |
| **Minerals (g/kg):** |  |  |
| Calcium carbonate | 12.5 | 12.5 |
| Potassium phosphate, monobasic | 6.86 | 6.86 |
| Potassium citrate, monohydrate | 2.48 | 2.48 |
| Sodium chloride | 2.59 | 2.59 |
| Potassium sulfate | 1.63 | 1.63 |
| Magnesium oxide | 0.85 | 0.85 |
| Ferric citrate | 0.21 | 0.21 |
| Zinc carbonate | 0.06 | 0.06 |
| Manganous carbonate | 0.02 | 0.02 |
| Cupric carbonate | 0.01 | 0.01 |
| Potassium iodate | 0.0004 | 0.0004 |
| Sodium selenate | 0.0004 | 0.0004 |
| Ammonium paramolybdate, tetrahydrate | 0.0003 | 0.0003 |
| Sodium meta-silicate, nonahydrate | 0.05 | 0.05 |
| Chromium potassium sulfate, dodecahydrate | 0.01 | 0.01 |
| Lithium chloride | 0.0006 | 0.0006 |
| Boric acid | 0.003 | 0.003 |
| Sodium fluoride | 0.002 | 0.002 |
| Nickel carbonate hydroxide, tetrahydrate | 0.001 | 0.001 |
| Ammonium meta-vanadate | 0.0002 | 0.0002 |
| Sucrose, fine ground | 7.73 | 7.73 |
|  |  |  |
| **Vitamins (g/kg):** | 7.73 | 7.73 |
| Niacin | 0.04 | 0.04 |

**Supplemental Table 1.** Diet composition.

**Supplemental Table 2.** Gene categories of interest for asthma and diet-induced asthma from DEG analysis (Figures 3C and D).

| **Category** | **Gene** | **Ref.^1^** | **Gene** | **Ref.^1^** |
| --- | --- | --- | --- | --- |
| Inflammation (pro) | M6PR  CXCL13  GFPT2  ZBTP16  FABP4  PdE4b  PCD5  AK2  S100a8  S100a9 | 1  2  3,4  5  6  7  8  9  10  10, 11 | RIPK3  CXCL12  CMTM4  TRIM56  ISOC1  DAB21P  CAMTA2  S100a11  AKAP5 | 12, 13  14  15  16  17  18  19  20  21 |
| Inflammation (anti) | FKBP5  TMEM66  Retnlg  STARD7  MAK14  MMP8 | 22  23  24  25  26  27 |  |  |
| Smooth muscle contraction | Mt2  PdE4b  Map3k6  Myl3  ACTA2  PPP2CB (activated by sphingolipids)  PIK3C2 | 28  29  30  31, 32  33  34, 35  36 |  |  |
| Smooth muscle proliferation | PRX2 | 37 | ZNF260 (inhibits)  TRABD2B (inhibits)  ARHGAP1 | 38  39, 40  41 |
| Airway remodeling | B6N  M6P | 42  43 |  |  |
| Lipid metabolism | ACOXL  FABP4  DLD | 32  6  44 |  |  |

^1^ Reference related to asthma

^2^ Only known for cardiac muscle

**Supplemental References**

1. Green, T.D., Crews, A.L., Park, J., Fang, S. & Adler, K.B. Regulation of mucin secretion and inflammation in asthma: a role for MARCKS protein? *Biochim Biophys Acta* **1810**, 1110-1113 (2011).

2. Baay-Guzman, G.J. *et al.* Role of CXCL13 in asthma: novel therapeutic target. *Chest* **141**, 886-894 (2012).

3. Zhang, Y. *et al.* The ORMDL3 Asthma Gene Regulates ICAM1 and has Multiple Effects on Cellular Inflammation. *Am J Respir Crit Care Med* (2018).

4. Al-Mukh, H. *et al.* Lipopolysaccharide Induces GFAT2 Expression to Promote O-Linked β-N-Acetylglucosaminylation and Attenuate Inflammation in Macrophages. *J Immunol* **205**, 2499-2510 (2020).

5. Feng, X. *et al.* iNKT cells with high PLZF expression are recruited into the lung via CCL21-CCR7 signaling to facilitate the development of asthma tolerance in mice. *Eur J Immunol* **51**, 414-432 (2021).

6. Ge, X.N. *et al.* FABP4 regulates eosinophil recruitment and activation in allergic airway inflammation. *Am J Physiol Lung Cell Mol Physiol* **315**, L227-l240 (2018).

7. Tsukiji, N. *et al.* Platelets play an essential role in murine lung development through Clec-2/podoplanin interaction. *Blood* **132**, 1167-1179 (2018).

8. Diao, X., Wang, J., Zhu, H. & He, B. Overexpression of programmed cell death 5 in a mouse model of ovalbumin-induced allergic asthma. *BMC Pulmonary Medicine* **16**, 149 (2016).

9. Picher, M. & Boucher, R.C. Human airway ecto-adenylate kinase. A mechanism to propagate ATP signaling on airway surfaces. *J Biol Chem* **278**, 11256-11264 (2003).

10. Kim, D.H. *et al.* Suppressive effects of S100A8 and S100A9 on neutrophil apoptosis by cytokine release of human bronchial epithelial cells in asthma. *Int J Med Sci* **17**, 498-509 (2020).

11. Quoc, Q.L. *et al.* S100A9 in adult asthmatic patients: a biomarker for neutrophilic asthma. *Experimental & Molecular Medicine* **53**, 1170-1179 (2021).

12. Oikonomou, N. *et al.* Airway epithelial cell necroptosis contributes to asthma exacerbation in a mouse model of house dust mite-induced allergic inflammation. *Mucosal Immunol* **14**, 1160-1171 (2021).

13. Zhang, H., Ji, J., Liu, Q. & Xu, S. MUC1 downregulation promotes TNF-α-induced necroptosis in human bronchial epithelial cells via regulation of the RIPK1/RIPK3 pathway. *J Cell Physiol* **234**, 15080-15088 (2019).

14. Bignold, R. *et al.* Chemokine CXCL12 drives pericyte accumulation and airway remodeling in allergic airway disease. *Respiratory Research* **23**, 183 (2022).

15. Bona, A. *et al.* MARVEL-domain containing CMTM4 affects CXCR4 trafficking. *Mol Biol Cell*, mbcE22050152 (2022).

16. Tsuchida, T. *et al.* The ubiquitin ligase TRIM56 regulates innate immune responses to intracellular double-stranded DNA. *Immunity* **33**, 765-776 (2010).

17. Kushnareva, Y. *et al.* Functional Analysis of Immune Signature Genes in Th1* Memory Cells Links ISOC1 and Pyrimidine Metabolism to IFN-γ and IL-17 Production. *The Journal of Immunology*, ji2000672 (2021).

18. Anzalone, G. *et al.* Cigarette smoke affects the onco-suppressor DAB2IP expression in bronchial epithelial cells of COPD patients. *Scientific reports* **9**, 15682-15682 (2019).

19. Rastogi, D. *et al.* CDC42-related genes are upregulated in helper T&#xa0;cells from obese asthmatic children. *Journal of Allergy and Clinical Immunology* **141**, 539-548.e537 (2018).

20. Xia, C., Braunstein, Z., Toomey, A.C., Zhong, J. & Rao, X. S100 Proteins As an Important Regulator of Macrophage Inflammation. *Front Immunol* **8**, 1908 (2017).

21. Harb, H. *et al.* A regulatory T cell Notch4-GDF15 axis licenses tissue inflammation in asthma. *Nature immunology* **21**, 1359-1370 (2020).

22. Kelly, M.M. *et al.* Corticosteroid-induced gene expression in allergen-challenged asthmatic subjects taking inhaled budesonide. *Br J Pharmacol* **165**, 1737-1747 (2012).

23. Wang, Y.M. *et al.* Store-operated calcium entry-associated regulatory factor regulates airway inflammation and airway remodeling in asthma mice models. *Am J Physiol Lung Cell Mol Physiol* **321**, L533-l544 (2021).

24. Lee, M.R. *et al.* Retnla overexpression attenuates allergic inflammation of the airway. *PLoS One* **9**, e112666 (2014).

25. Yang, L. *et al.* Haploinsufficiency for Stard7 is associated with enhanced allergic responses in lung and skin. *J Immunol* **194**, 5635-5643 (2015).

26. Xia, T., Ma, J., Sun, Y. & Sun, Y. Androgen receptor suppresses inflammatory response of airway epithelial cells in allergic asthma through MAPK1 and MAPK14. *Hum Exp Toxicol* **41**, 9603271221121320 (2022).

27. Quintero, P.A., Knolle, M.D., Cala, L.F., Zhuang, Y. & Owen, C.A. Matrix metalloproteinase-8 inactivates macrophage inflammatory protein-1 alpha to reduce acute lung inflammation and injury in mice. *J Immunol* **184**, 1575-1588 (2010).

28. Yin, L.M. *et al.* Transgelin-2 as a therapeutic target for asthmatic pulmonary resistance. *Sci Transl Med* **10** (2018).

29. Krymskaya, V.P. & Panettieri, R.A., Jr. Phosphodiesterases regulate airway smooth muscle function in health and disease. *Curr Top Dev Biol* **79**, 61-74 (2007).

30. Gerthoffer, W.T. *et al.* Activation of MAP kinases in airway smooth muscle. *Am J Physiol* **272**, L244-252 (1997).

31. Gao, N. *et al.* Signaling through myosin light chain kinase in smooth muscles. *J Biol Chem* **288**, 7596-7605 (2013).

32. Zhu, Z. *et al.* Shared genetic and experimental links between obesity-related traits and asthma subtypes in UK Biobank. *J Allergy Clin Immunol* **145**, 537-549 (2020).

33. Moiseenko, A. *et al.* Origin and characterization of alpha smooth muscle actin-positive cells during murine lung development. *Stem Cells* **35**, 1566-1578 (2017).

34. Dobrowsky, R.T., Kamibayashi, C., Mumby, M.C. & Hannun, Y.A. Ceramide activates heterotrimeric protein phosphatase 2A. *Journal of Biological Chemistry* **268**, 15523-15530 (1993).

35. Takai, A. *et al.* Protein phosphatases 1 and 2A and their naturally occurring inhibitors: current topics in smooth muscle physiology and chemical biology. *The Journal of Physiological Sciences* **68**, 1-17 (2018).

36. Sarker, M.A.K. *et al.* Class II PI3Ks α and β Are Required for Rho-Dependent Uterine Smooth Muscle Contraction and Parturition in Mice. *Endocrinology* **160**, 235-248 (2019).

37. Jones, F.S., Meech, R., Edelman, D.B., Oakey, R.J. & Jones, P.L. Prx1 controls vascular smooth muscle cell proliferation and tenascin-C expression and is upregulated with Prx2 in pulmonary vascular disease. *Circ Res* **89**, 131-138 (2001).

38. Lepore, J.J., Cappola, T.P., Mericko, P.A., Morrisey, E.E. & Parmacek, M.S. GATA-6 Regulates Genes Promoting Synthetic Functions in Vascular Smooth Muscle Cells. *Arteriosclerosis, Thrombosis, and Vascular Biology* **25**, 309-314 (2005).

39. Grainger, S. & Willert, K. Mechanisms of Wnt signaling and control. *Wiley Interdiscip Rev Syst Biol Med*, e1422 (2018).

40. Danielewicz, H. *et al.* Pet ownership in pregnancy and methylation pattern in cord blood. *Genes Immun* **22**, 305-312 (2021).

41. Loirand, G. & Pacaud, P. Involvement of Rho GTPases and their regulators in the pathogenesis of hypertension. *Small GTPases* **5**, 1-10 (2014).

42. Huang, J., Olivenstein, R., Taha, R., Hamid, Q. & Ludwig, M. Enhanced proteoglycan deposition in the airway wall of atopic asthmatics. *Am J Respir Crit Care Med* **160**, 725-729 (1999).

43. Xing, Y.M., Li, P.S. & Liu, Y. 1,25-(OH)2D3 participates and modulates airway remodeling by reducing MGP and TGF-β1 expression in TNF-α-induced airway smooth muscle cells. *Adv Clin Exp Med* **31**, 151-155 (2022).

44. Ijpma, G. *et al.* Intrapulmonary airway smooth muscle is hyperreactive with a distinct proteome in asthma. *Eur Respir J* **56** (2020).
